# Multiple Dural Venous Sinus Thromboses in a Young Female Presenting With Refractory Headaches

**DOI:** 10.7759/cureus.91894

**Published:** 2025-09-09

**Authors:** Ajith S Bharadwaj, Karson Munson, Moneeb Mustafa, Prathapraju Poloju

**Affiliations:** 1 Internal Medicine, Edward Via College of Osteopathic Medicine, Alexandria, USA; 2 Internal Medicine, CHRISTUS St. Frances Cabrini Hospital, Alexandria, USA; 3 Internal Medicine, Rapides Regional Medical Center, Alexandria, USA

**Keywords:** antiphospholipid antibody syndrome (aps), dural venous sinus thrombosis, hemato-oncology, internal medicine (general medicine), vascular neurology

## Abstract

Dural venous sinus thrombosis (DVST) is a condition that affects the venous sinuses of the brain. Clotting in this vasculature can lead to the presentation of headache, seizures, stroke-like symptoms, and other neurologic deficits. Proper investigation requires clinicians to understand and explore Virchow’s triad and the elements that lead to a thrombus.

Herein is the case of a 26-year-old female with a history of refractory headaches attributed to migraines who presented to the ER with altered mentation. Sudden unexplained seizure-like activity presented, which warranted further workup. Further investigation by analyzing serum antibodies, inheritable causes of hypercoagulability, screening for infections, and using advanced imaging like magnetic resonance venography confirmed the presence of multiple DVSTs in the peripheral straight, sagittal, and occipital sinuses. Anticoagulant heparin was administered during hospitalization, and once treated, the patient was discharged to follow up on an outpatient basis with apixaban for continuity of care.

## Introduction

Dural venous sinus thrombosis (DVST) is an incredibly rare vascular pathology that affects three to five individuals per one million adults [1]. Similar to how other clots form, and through the application of Virchow’s triad, we can understand that hypercoagulability, injury, and stasis of blood flow can contribute to a thrombus. Other hypercoagulable states as a result of malignancy, pregnancy, infection, and oral contraceptive use may also contribute to thrombus formation [2]. Additionally, females are more likely to be affected by DVSTs than males by a ratio of 3:1 and were more likely to be afflicted by DVSTs at a much younger age (median 34 years old for females and median 42 years old for males) [3,4]. DVSTs present with nonspecific symptoms. Patients may present with headaches with or without emesis, visual changes, papilledema, seizures, focal deficits, mental status changes, decreased level of consciousness, and other signs of encephalopathy [2].

To understand the symptomatology of DVSTs, it is important to understand the function of the dural venous sinuses. Dural sinuses, also known as cerebral sinuses, are vascular structures that drain deoxygenated venous blood within the cranial vault to the internal jugular vein for recirculation into systemic circulation [5]. There are seven major sinuses within the cranial vault: the straight sinus, the superior and inferior sagittal sinuses, and the transverse, sigmoid, petrosal, and cavernous sinuses. Among these sinuses, the superior sagittal sinus also has the function of draining cerebrospinal fluid (CSF) [5]. Obstruction of the vasculature, as a result of thrombus-like formation in DVSTs, or as a result of mass effect from tumors, can lead to increased intracranial pressure and various neurologic deficits due to impaired CSF absorption. This can present in a patient as headaches, papilledema, and altered mental status [2]. Additionally, blood flow obstruction through the sinuses may also lead to cerebral parenchymal lesions, such as hemorrhagic stroke, the culmination of vastly elevated venous pressures [6].

We report a case of a 26-year-old female who presented with non-specific symptoms consisting of headaches, altered mental status, and seizure with recently negative head CT scans. The patient was confirmed to have widespread thromboses of the dural venous sinuses.

## Case presentation

A 26-year-old female presented to the emergency department with chronic debilitating headaches for the past 3 months. The patient described these headaches as a bifrontal pressure-type headache that had been persistent. Prior to this admission, she had visited three other emergency departments with the same complaints and was prescribed sumatriptan as an abortive pharmacologic therapy. The patient noted that this pain was associated with occasional blurry vision, which resolved spontaneously. She also alleged that the headaches caused significant nausea, sonophobia, and photophobia. The headaches were alleviated with lying down and were not associated with any focal weakness or numbness. Additionally, the patient did not present with any neck stiffness, fevers, or other rapid-onset symptoms. Characteristically, the headache presented with migraine symptomatology, although the classic aura symptoms were not noted. A diagnosis of migraine was best supported due to head CT scans from the previous week that were negative for any acute pathology.

The patient’s mother was present during the initial history. The mother noted that the patient had an altered mental status with waxing and waning levels of consciousness. While taking the initial history, the patient was arousable, able to answer questions, but did have a reduced level of consciousness. However, re-evaluation after some time revealed a well-oriented, awake, and alert patient. Social history was unrevealing for alcohol use or illicit drug use, and the patient indicated that they had never smoked. Physical exam revealed an obese 26-year-old female with an unrevealing physical exam. No signs of photophobia, visual changes, head trauma, fever, or edema were present. Kernig and Brudzinski signs were negative, and there were no motor or sensory deficits present. Pertinent lab findings included a WBC of 14.9 cells/µL, prolactin 46.3 ng/mL, and a glucose level of 168 mg/dL. Urine drug screen, urinalysis, and HIV testing were negative. The CT scan of the head and neck without contrast was performed, and both were negative for any acute processes. The chest radiograph was also negative for any pathologic changes. During further assessment for pain management and fall risk, the patient began demonstrating seizure-like activity with eyes rolling superiorly and with occasional crossing. The patient also had an episode of emesis during this time. She was started on levetiracetam 1,000 mg and lorazepam 1 mg for seizure control. A loading dose of vancomycin 2,500 mg was given due to a positive blood culture with Gram-positive cocci. Supportive treatments were provided, as needed, for pain and nausea. Neurology and Internal Medicine were consulted for her admission and continuation of management.

**Figure 1:**
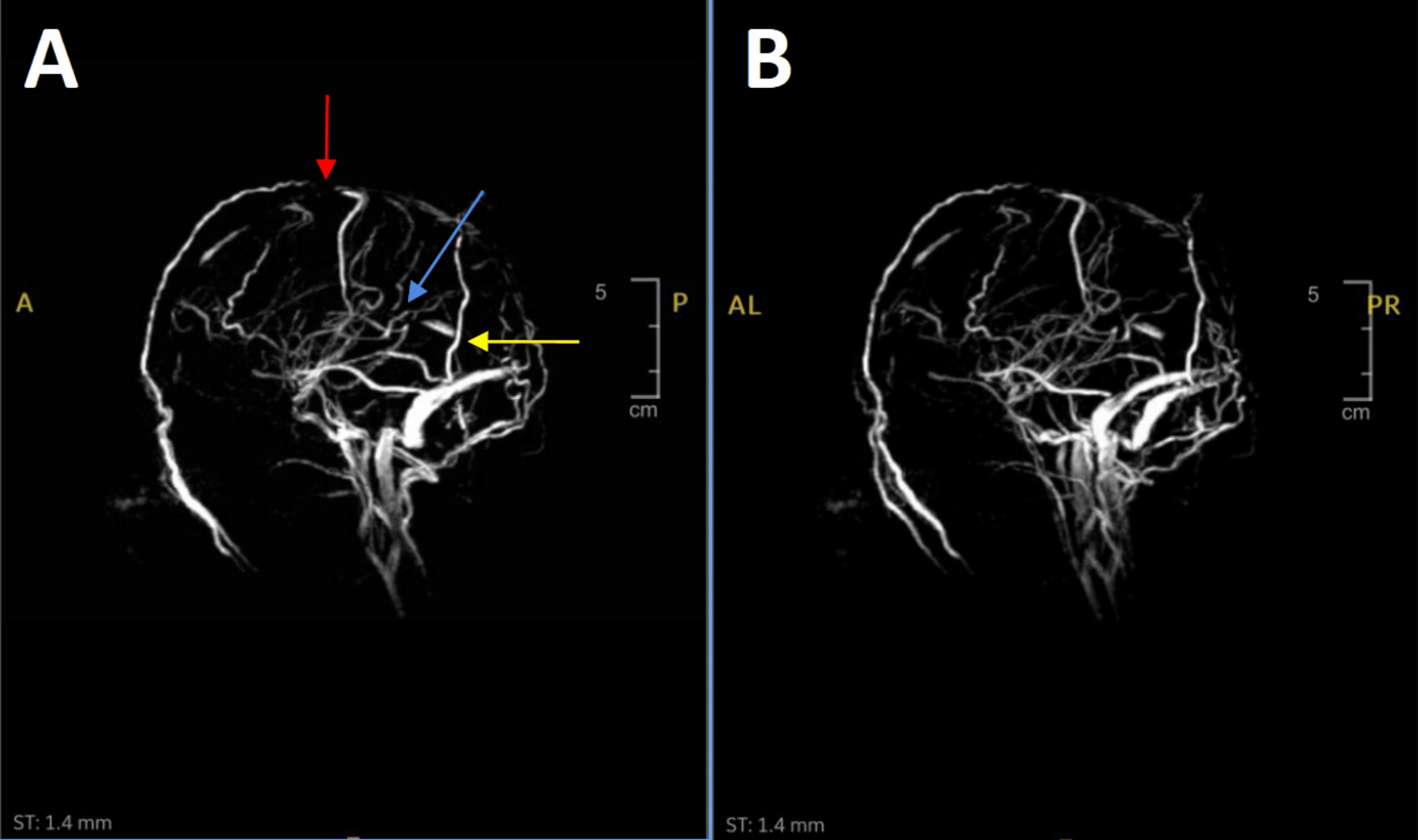
MRV imaging of the brain showcasing opacifications, consistent with multiple areas of thrombosis Notable opacification and irregularity can be seen in the peripheral straight sinus, superior sagittal sinus, and inferior sagittal sinus, consistent with thrombosis. Panel A is annotated to show these findings. The red arrow highlights the location of the thrombus at the superior sagittal sinus. The blue arrow indicates the presence of a thrombus at the inferior sagittal sinus, and the yellow arrow marks the position of another at the straight sinus. Panel B reveals a clearer picture of the opacification at the straight sinus. MRV: MR venography

Neurology consultation recommended a brain MRI with and without contrast, an MR angiography (MRA) of the head without contrast, and an MR venography (MRV) without contrast to rule out venous sinus thrombosis. An EEG was ordered to further evaluate seizure activity. Finally, a spinal tap was ordered with opening and closing pressures and fluid analysis. Images of the MRV are shown in Figure 1.

The MRV showed incomplete opacification and diffuse irregularity of the superior sagittal sinus and partial opacification of the inferior sagittal sinus, with truncation of flow within the peripheral straight sinus. This finding, combined with the patient’s symptoms, was concerning for a dural venous sinus thrombosis. The internal cerebral veins, transverse sinuses, and jugular bulbs were unobstructed. The MRI and MRA did not demonstrate any significant abnormalities. The patient’s EEG showed a normal electroencephalogram during the awake, drowsy, and sleep states. The lumbar puncture revealed an elevated opening pressure of 33 cm H2O and a closing pressure of 12 cm H2O. A specimen was obtained and sent for analysis. The CSF findings were consistent with viral meningitis, with a glucose level of 63 mg/dL and a WBC count of 125 cells/µL, with 100% lymphocytes.

During her hospital stay, the patient had episodes of bradycardia, prompting a Cardiology consult. The ECG showed a normal sinus rhythm with episodes of premature atrial complexes (PACs) and premature ventricular complexes (PVCs) with compensatory pausing. No episodes of AV nodal disease or arrhythmias were noted. The patient had a positive chronotropic response. A 2D echo was ordered to rule out a cardiac origin for the patient’s symptoms. The echocardiogram findings were normal, and thus a cardiac origin of the patient’s symptoms was eliminated in lieu of a neurologic or coagulopathic origin.

In addition to supportive care for the viral meningitis, the patient was also started on heparin injections, 25,000 units, for treatment of the DVSTs to improve the patient’s symptoms and prevent future complications. Upon further questioning, the patient denied any previous blood clots. However, the patient’s mother stated that there are “problems with blood clots” on the mother’s side of the family. It was also revealed that the patient’s father had a myocardial infarction at the age of 52. Given the patient’s presentation and her family history, Hematology-Oncology was consulted to investigate possible coagulopathic disorders that led to this patient’s thrombosis. To assess for a thrombophilic disorder, lupus anticoagulant, Factor V Leiden mutation, anti-cardiolipin, anti-beta 2 glycoprotein, EBV IgM, and CMV tests were ordered.

Over the course of her hospital stay, the patient improved clinically while being on heparin. A repeat MRV with and without contrast showed significant improvement and stabilization of the DVST. The patient’s presenting symptoms of headaches and diplopia were also improved. A full thrombophilia panel was obtained, which included testing for Factor V Leiden, prothrombin gene mutation, antithrombin III, protein C and S deficiencies, and lupus anticoagulant, all of which were negative. However, the patient tested positive for the anti-cardiolipin antibody test, with an elevated immunoglobulin M (IgM) of 43 MPL (normal ≤ 12). A positive anticardiolipin test can have a possible association with antiphospholipid syndrome, a condition where the body is predisposed to clotting. Further, these antibodies are often detected in patients with autoimmune disorders such as systemic lupus erythematosus. This finding prompted the need for further investigation in an outpatient setting. Therefore, the patient was discharged but scheduled for a follow-up with her primary care physician in five days and scheduled for a follow-up with the Hematologist-Oncologist in a few weeks to discuss the thrombophilia test results and adjust treatment as needed. The importance of the follow-up appointments was explained in great detail. Levatiracetam was continued to prevent future seizure activity. The patient was switched from heparin to apixaban due to its oral administration, predictable effect, and reduced need for monitoring. Due to the positive blood culture on admission, the patient was discharged home with PO antibiotics to continue. The patient was advised against traveling, and the possible side effects of the medication were explained. While the patient’s symptoms had improved from where they were on admission, the patient still suffered from blurry vision, likely a consequence of the DVSTs. Therefore, an eye patch and a follow-up appointment with an ophthalmologist were recommended. Further management was dependent on confirmatory tests for antiphospholipid syndrome and systemic lupus erythematosus in an outpatient setting. However, the patient’s initial presenting symptoms, such as the debilitating headaches, inflammatory process, altered mental status, and seizure-like activity, were all improved with the concomitant use of levetiracetam, vancomycin, and heparin injections to stabilize the DVSTs found on MRV and its subsequent complications.

## Discussion

The location within the cranial vault and pathophysiologic effects make DVSTs intrinsically medically significant. Imaging, in particular, is essential for the early treatment of DVST, as earlier detection is associated with better outcomes and for the prevention of sequelae [7]. In a patient with any presentation concerning DVST, the recommended neuroimaging includes the MRI and MR venography (MRV) or CT and CT venography (CTV) of the brain [8]. The MRI and CT scans best demonstrate the absence of flow and intraluminal venous thrombus formation, which is essential for the confirmation of diagnosis. However, if not readily apparent in the MRI and CT, an MRV and CTV can demonstrate an absence of flow, helping confirm the diagnosis [9]. In our case, a standard CT scan did not capture the image of the DVST; therefore, an MRV was ordered due to clinical suspicion, confirming the diagnosis.

There are also some interesting patterns where DVSTs are commonly found. A study from 2014 suggested that the most common sites of DVSTs include the transverse (56%) and sagittal sinuses (51%) [10]. Another study found similar findings among 160 patients with DVSTs that suggested the superior sagittal sinus (65%) and transverse sinuses (60.5%) as being the most common locations for thromboses [11]. The same study suggested that in the majority of patients, DVSTs often involve multiple sinuses (71.2%), which is similar to the findings in our reported case [11,12]. As mentioned in the introduction, there are seven major cerebral venous sinuses in the human body. They each circulate venous blood from various parts of the brain, contributing to the regulation of circulation. The sagittal sinus is of particular importance given its reception of CSF from arachnoid granulations in addition to venous blood. Understanding how a thrombus in such an area can impact CSF absorption and cause venous obstruction, consequently elevating ICP and venous pressures, can help clinicians reason why this may cause focal neurologic symptoms, seizures, and headaches. Having a better understanding of the effects of DVST and common locations of thrombus formation can help clinicians have the necessary suspicions to pursue further workup.

As mentioned previously, major risk factors for dural venous sinus thrombosis can be explained by Virchow’s triad: venous stasis, endothelial injury, and a hypercoagulable state. Venous stasis as a result of dehydration, mass effect, etc., can be caused by decreased flow and obstruction. Direct trauma or infections, such as meningitis, as seen in our patient, can contribute to an inflammatory response, disrupting the integrity of the cerebral vasculature (endothelial injury). A hypercoagulable state contributed by genetic or acquired conditions like pregnancy, antithrombin III, proteins C and S deficiency, Factor V Leiden, and antiphospholipid syndrome (APS) can contribute to clot formation [13]. As a whole, our patient had confirmed viral meningitis and serum analysis for possible APS coagulopathy with the presence of IgM anti-cardiolipin. These coupled factors may have led to increased susceptibility to clot formation, leading to the condition our patient presented with. This topic will be further explored in the discussion. A thorough understanding of Virchow’s triad is essential for acquiring a proper patient history and workup. Having knowledge of the various elements that can lead to thrombosis, as described by Virchow’s triad, can help investigate potential causes of coagulopathy, aiding proper treatment and reducing future hospitalizations. This involves a thorough laboratory investigation of blood and serum, a thrombophilia workup, assessing for potential infections, and imaging to assess for any acute or pathologic causes [13].

Fortunately, this patient had a favorable outcome with treatment. By the time of her discharge, her major symptoms had improved significantly. However, she was not completely back to normal. She had lingering blurry vision, indicating that her intracranial pressure was still stabilizing, or perhaps was suffering a cranial nerve palsy resulting from the thrombosis. This is why outpatient follow-up with ophthalmology was recommended. Residual symptoms are common but often resolve over time due to the restoration of blood flow. Reassuringly, the repeat MRV toward the end of her hospital stay already showed improvement in the sinus flow. Perhaps the most crucial part of her treatment plan is the use of anticoagulation. Anticoagulation is typically continued for a significant amount of time in a dural venous sinus thrombosis. Current guidelines recommend that anticoagulation be continued for three to six months in provoked cases, such as if an infection is the cause [14]. Moreover, anticoagulation should be continued for 6 to 12 months in unprovoked cases, with definitive therapy recommended if an underlying thrombophilic condition is present [15]. In this patient, if antiphospholipid syndrome is confirmed, she would likely require long-term anticoagulation therapy. Apixaban was chosen for outpatient management. While low-molecular-weight heparin or warfarin have traditionally been used for cerebral venous thrombosis, newer evidence suggests that direct oral anticoagulants (DOACs) like apixaban are effective and safe as well [16]. A recent multicenter study also showed that apixaban may be a safe alternative to warfarin in patients with cerebral venous thrombosis, with similar outcomes between the two [17]. Patients with antiphospholipid syndrome are the exception because warfarin remains the gold standard treatment if high-risk antibodies are present [17]. Given that our patient’s antiphospholipid syndrome status was not confirmed, using apixaban was a reasonable decision in the interim. It provided a convenient, fixed-dose regimen for the patient and avoided the need for injections or frequent lab draws. As emphasized in the discharge plan, making sure that the patient adhered to the treatment plan and follow-up appointments was of paramount importance. She was counseled on medication adherence and lectured on the avoidance of high-risk activities, such as long travel, which may increase the risk of a clot. This kind of counseling is worth mentioning because young patients may underestimate the importance of continuing treatment, especially when discharged from the hospital and feeling better. In a patient with antiphospholipid syndrome or a DVST, non-compliance can have devastating consequences, such as clot extension or recurrence. The continuation of levetiracetam was precautionary. Seizures in DVST are often isolated to the acute phase, but recurrence can occur; therefore, continuing anticonvulsant medication for a short time is often indicated. It should be noted, though, that in this specific patient, given her normal EEG and no evident cortical damage, long-term anticonvulsants may not be necessary. If the patient remains seizure-free after a few months, tapering off the medication may be an option.

The patient’s CSF analysis was consistent with viral meningitis, which likely contributed to a transient pro-inflammatory state. While it is rare, viral infections such as enteroviruses and herpesviruses have been shown to contribute to the development of cerebral venous thrombosis [18]. For this specific case, viral meningitis may have acted as a precipitating factor for the dural venous sinus thromboses in a patient with a possible underlying predisposition. In addition, the patient’s initial blood culture tested positive for Gram-positive cocci. The most likely cause of this culture result was a skin contaminant. Despite this, the possibility of an underlying bacteremia could not be ignored, and hence the patient was treated empirically. While none of these infectious processes escalated or resulted in severe complications, supportive care and antibiotics had to be a focus, in addition to managing the patient’s thrombosis.

## Conclusions

The discussion of this case focuses on a rare scenario in which a presentation similar to a common condition, like migraines, may mask the possibly life-threatening diagnosis of a DVST. This case emphasizes that recurrent or refractory headaches in young patients may be misdiagnosed as migraine, especially with the absence of focal neurological signs or symptoms, and the importance of considering DVSTs in such a patient group. For this patient, the development of altered mental status and seizures prompted further investigation, leading to the correct diagnosis. The etiology of DVST is still not completely understood. At this stage of medical understanding, clinicians are urged to understand Virchow’s triad and how the three aspects described can contribute to thrombus formation. Patients who present with risk factors for thrombus formation who seek care with headache or nonspecific neurologic presentations should undergo a complete thrombophilia workup and specific neuroimaging modalities, such as MRV and CTV, in addition to a standard workup. Early detection of DVSTs can ensure improved outcomes and reduced occurrence of potential sequelae in patients.

This case emphasizes the importance of a thorough workup, including detailed history-taking, specific imaging, and procedures like a lumbar puncture. It also demonstrates the need to consider underlying hypercoagulable states in a young patient with a thrombosis, with antiphospholipid syndrome being the likely cause in this case. Prompt anticoagulation and supportive care are essential for providing an excellent recovery. The interconnection of neurology, radiology, hematology-oncology, and internal medicine was crucial in managing the complex nature of this case. This interdisciplinary coordination is essential for the management of the condition and successful outcomes. Lastly, this case ultimately demonstrates a successful outcome and serves as a reminder to physicians that not all that looks like a migraine is benign, and sometimes, the “zebra” diagnosis is real and requires further investigation.
